# Sleep disturbances and depression severity in patients with Parkinson's disease

**DOI:** 10.1002/brb3.967

**Published:** 2018-04-23

**Authors:** Daniel B. Kay, Jared J. Tanner, Dawn Bowers

**Affiliations:** ^1^ Department of Psychology Brigham Young University Provo UT USA; ^2^ Department of Clinical and Health Psychology University of Florida Gainesville FL USA

**Keywords:** insomnia, Parkinson's disease, short sleep duration, sleep vulnerability

## Abstract

**Objectives:**

Parkinson's disease (PD) is a multisystem movement disorder associated with sleep disturbance and depression. Sleep disturbances and depression severity share a bidirectional association. This association may be greater in individuals who are more vulnerable to the deleterious consequences of sleep disturbance and depression severity. We investigated whether the association between sleep disturbances and depression severity is greater in patients with PD than in matched controls (MC).

**Materials and Methods:**

The study sample (*N *=* *98) included 50 patients with idiopathic PD and 48 age‐, race‐, sex‐, and education‐matched controls. Sleep disturbances were assessed using self‐reported total sleep time (TST) on the Pittsburgh Sleep Quality Index, the sleep item on the Beck Depression Inventory, 2nd ed. (BDI‐II), and the Insomnia Severity Index total score. Depression severity was assessed using the BDI‐II total score, excluding the sleep item. Spearman's correlations, chi‐squared tests, and multiple regression were used to assess associations between sleep disturbances and depression severity in PD and MC. Fisher's *Z* transformation was used to test whether the association between sleep disturbances and depression severity was stronger in patients with PD.

**Results:**

Shorter TST, sleeping less than usual, and insomnia severity were associated with depression severity in the total sample, *r*_*s*_(94) = −0.35, *p *=* *.001; *r*_*s*_(71) = 0.51, *p *<* *.001; *r*_*s*_(78) = −0.47, *p *<* *.001; *r*_*s*_(98) = 0.46, *p *<* *.001, respectively. The association between shorter TST and depression severity was greater in patients with PD than it was in MC,* p < *.05.

**Conclusion:**

Short TST may be an important marker, predictor, or consequence of depression severity in patients with Parkinson's disease.

## 1 INTRODUCTION

Idiopathic Parkinson's disease (PD) is one of the most common neurodegenerative disorders, second only to Alzheimer's disease. Historically viewed as a motor disorder, PD is a complex, multisystem disorder associated with nonmotor symptoms including sleep changes and depression. Sleep disturbances are common, affecting about 60*%* of individuals with PD (Chahine, Amara, & Videnovic, 2016; Tandberg, Larsen, & Karlsen, 1998). Depression symptoms, predominantly mild, are also highly prevalent in patients with PD, affecting 14%–35% of patients (Reijnders, Ehrt, Weber, Aarsland, & Leentjens, 2008; Rutten et al., 2017).

In the general population, sleep disturbances, including self‐reported shorter total sleep time (TST) and insomnia complaints—self‐reported difficulty initiating or reinitiating sleep—are cross‐sectionally and prospectively associated with depression severity (Katz & McHorney, 2002; Nebes, Buysse, Halligan, Houck, & Monk, 2009; Ohayon, 1997; Ohayon & Roth, 2003; Tworoger, Lee, Schernhammer, & Grodstein, 2006; Varkevisser, Van Dongen, Van Amsterdam, & Kerkhof, 2007). Sleep disturbances including altered TST and insomnia are also associated with depression symptoms in PD (Avidan et al., 2013; Borek, Kohn, & Friedman, 2006; Gjerstad, Wentzel‐Larsen, Aarsland, & Larsen, 2007; Goetz, Wilson, Tanner, & Garron, 1987; Kumar, Bhatia, & Behari, 2002; Margis, Donis, Schonwald, & Rieder, 2010; Naismith, Hickie, & Lewis, 2010). Short TST and insomnia are overlapping but separate sleep problems that may be associated with depression through different pathophysiological mechanisms. Investigating the association between different types of sleep disturbances and depression severity in patients with PD may help elucidate their pathophysiology.

Some individuals experience greater depression severity following sleep deprivation than others (Van Dongen, Baynard, Maislin, & Dinges, 2004). Proposed vulnerability factors for experiencing sleep‐related depression include age, genetics, health status, and cognitive status (Drummond et al., 1999). We and others have proposed that sleep disturbances may lead to greater sleep‐related impairments in patients with neurodegenerative diseases than individuals who do not have these disorders (Seugnet, Galvin, Suzuki, Gottschalk, & Shaw, 2009). In other words, these patients may be more vulnerable to the negative impact of sleep disturbances. Preliminary research lends support to this hypothesis. In a Drosophila model of PD, sleep deprivation resulted in greater short‐term memory impairments in PD animals compared to controls (Seugnet et al., 2009). Although human research on this hypothesis is sparse, one study found sleep disturbances are both more common and severe among patients with PD than among healthy older adults (Tandberg et al., 1998). These results may suggest patients with PD find sleep disturbances more distressing or impairing than the general adult population. Whether the association between sleep disturbances and depression severity is greater in patients with PD remains unclear. In this study, we explored the associations between different types of self‐reported sleep disturbances (differences in TST and insomnia severity) and depression symptoms in a sample of patients with PD and in MC. We hypothesized that sleep disturbances would be more strongly associated with depression symptoms in patients with PD than in MC.

## 2 MATERIALS AND METHODS

### 2.1 Participants

Participants included 50 patients with idiopathic PD and 48 neurologically healthy older adults matched for age, race, sex, and education. Participants with PD were recruited from the University of Florida (UF) Center for Movement Disorders and Neurorestoration (CMDN) during their routine visits. Clinical diagnosis of idiopathic PD was determined by a movement disorders specialist based on the United Kingdom Parkinson’s Disease Society Brain Bank clinical diagnostic criteria using the Unified Parkinson Disease Rating Scale (UPDRS) (Hughes, Daniel, Kilford, & Lees, 1992; Fahn & Elton, 1987). Eligible patients had to demonstrate marked improvement in motor symptoms in response to initiation of dopaminergic therapy, based on the motor subscore of the UPDRS following administration of levodopa. All patients with PD were stabilized on medications for at least 3 months before participation and were in the early to mid‐stages of disease severity based on Hoehn–Yahr staging of 3 or less (Hoehn & Yahr, 1967).

Exclusion criteria were (i) inability to provide informed consent; (ii) less than 8th grade education; (iii) evidence of significant neurologic disturbance other than PD (toxic exposure, tumors, encephalitis, epilepsy, stroke in the past year, head injury with loss of consciousness > 24 hr, etc.); (iv) other clinically significant medical problems such as metastatic cancer, human immunodeficiency virus, blindness/deafness, liver or renal disease, severe anemia, myocardial infarction (< 6 months), congestive heart failure (functional stage > 3), and so forth; (v) severe psychiatric disease including significant history of substance abuse or current history of untreated alcohol or substance abuse, severe major depressive disorder determined by a cutoff score > 19 on the Patient Health Questionnaire‐9 (Kroenke, Spitzer, & Williams, 2001), bipolar disorder, schizophrenia, or post‐traumatic stress disorder; (vi) reduced cognitive status based on scores less than 25 on the Mini‐Mental State Exam (MMSE); (vii) inability to read and understand English; (viii) brain surgery (deep brain stimulation surgery or pallidotomy). Additional exclusion criteria related to sleep included (i) self or bed partner report of symptoms on the Pittsburgh Sleep Quality Index (PSQI), diagnosis, or treatment for obstructive sleep apnea (OSA), restless leg syndrome (RLS), or periodic limb movement disorder (PLMD) or (ii) lack of bed partner who was able to document the absence of OSA, RLS, and PLMD. There were no exclusions for medication use.

Disease‐specific information about the PD participants was obtained from an IRB‐approved clinical research database maintained by the UF CMDN. Obtained information included standard clinical measures for staging the severity of motor symptoms and disease course: UPDRS, a modified Hoehn–Yahr scale, and the Schwab and England Activities of Daily Living scale, duration and onset of motor symptoms, laterality of initial symptoms, levodopa equivalent dose (LED), medications, and PD symptom subtype (tremor predominant or akinetic‐rigid) (Hoehn & Yahr, 1967; Schwab & England, 1969). Mean (*M*) and standard deviation (*SD*) for PD duration was 7.8 (4.9) years, levodopa equivalent dose was 756.0 (493.6) mg, and Hoehn–Yahr stage was 1.8 (1.1). The majority of patients were tremor predominant (70%).

Control participants were recruited through interested patient spouses, newspaper advertisements, community fliers, local aging communities, and mailers. Individuals were excluded for the above exclusion criteria of the PD group or if they had a history of a movement disorder.

Table 1 shows the demographic characteristics of the PD and MC groups. The patients with PD were predominantly men (76%), 50 to 80 years of age (*M *=* *67 years), primarily white (96%), well‐educated (*M *=* *16 years), married (86%), and not employed (80%) at the time of study participation. Matched controls were predominantly men (77%) and were in their mid‐late 60 s in age (*M *=* *67 years), white (96%), well‐educated (*M *=* *17 years), married (77%) and not employed (73%). Controls and patients with PD did not differ on any of these demographic variables.

**Table 1:** Demographic, health, and sleep features in patients with Parkinson's disease (PD) compared to matched controls (MC)

Feature	MC (*n *=* *48)	PD (*n *=* *50)	*df*	*F*/χ^2^/Z	*p*
Age, years	67.5 (7.5)	67.4 (7.2)	96	<0.01	.935
Education, years	16.6 (3.0)	16.0 (3.0)	96	0.85	.359
Female gender, *n*	11	12	1	0.02	.899
White, *n*	46	48	2	<0.01	.999
MMSE score	29 [28,30]	29 [28,30]	–	0.28	.778
PSQI total score	5 [3, 7]	6 [4, 10]	–	2.27	.023*
TST, PSQI	420 [387.465]	420 [360,450]	–	1.20	.231
PSQI >5, *n*(%)	18 (38%)	29 (58%)	1	4.12	.042*
Depression severity (BDI‐II)	2 [1,4]	6.5 [3,12]	–	5.35	<.001***
More sleep (BDI‐II item 16a), *n*	4	15	1	9.51	.002**
Less sleep (BDI‐II item 16b), *n*	11	15	1	2.82	.093
ISI total score	4 [1,7]	6 [4,11]	–	2.57	.010*
Average medical conditions	4 [2,6]	4 [3,6]	–	0.03	.977
Average number of medications	2.5 [1,4]	4 [2,6]	–	2.96	.003**
Antidepressant, *n*	1	14	1	12.69	<.001***
Sleep aid, *n*	4	16	1	8.45	<.001***
Disease duration, years	–	7.8 (4.9)	–	–	–
Levodopa equivalent dose	–	756.0 (493.6)	–	–	–
Hoehn–Yahr stage	–	1.8 (1.1)	–	–	–
Tremor predominant subtype, %	–	70	–	–	–

BDI‐II, Beck Depression Inventory 2nd ed.; ISI, Insomnia Severity Index; MC, Matched controls; Mean (Standard Deviation), Median [Standard Deviation]; MMSE, Mini‐Mental Status Exam; PD, Parkinson's disease; Pittsburgh Sleep Quality Index (PSQI); TST, total sleep time. *p <.05, **p <.01, ***p <.001.

### 2.2 Procedures

This study was conducted with approval from the UF Institutional Review Board and in accordance with The Code of Ethics of the World Medical Association (Declaration of Helsinki) for experiments involving humans. Participants provided written informed consent prior to completing study procedures. Participants completed screening, a clinical sleep interview, and various questionnaires related to sleep, depression, and quality of life in a clinical testing room at UF or in participants’ homes. All patients with PD were assessed in the ON state (i.e., while medicated). A modified clinical sleep interview was used to assess sleep history, psychosocial functioning, medical conditions, medications, and mental health.

### 2.3 Measures

#### 2.3.1 Total sleep time

Item number 4 of the PSQI was used to assess average TST participants typically obtained per night over the past month. This item has been used in numerous studies as a measure of subjective TST (e.g., Nebes et al., 2009). Total sleep time, in minutes, was used as an independent variable in this study.

#### 2.3.2 Sleep changes

Item 16 of the Beck Depression Inventory, 2nd ed. (BDI‐II) was used to assess changes in sleep over the past 2 weeks (Beck, Steer, & Carbin, 1988). Responses to 16a (sleeping more than usual) or 16b (sleeping less than usual) >0 were recorded as indicating sleeping more or sleeping less than usual. This item was used to derive two variables: (i) sleeping more than usual or no change and (ii) sleeping less than usual or no change. Thus, for each of these variables, the change in sleep was compared to those who reported no change in sleep.

#### 2.3.3 Insomnia symptom severity

The Insomnia Severity Index (ISI) is a seven‐item questionnaire used to assess sleep quality and insomnia severity over the previous 2 weeks (Bastien, Vallieres, & Morin, 2001). The ISI is a continuous measure with scores ranging between 0 and 28. The independent variable of interest was total ISI score.

#### 2.3.4 Depression symptom severity

The BDI‐II, a well‐validated 21‐item self‐report inventory, was used in this study to quantify the severity of depression symptoms. The BDI‐II is widely used to assess depression in older adult samples and a Movement Disorders Society task force recommended the BDI as a valid continuous measure of depression severity in PD (Schrag et al., 2007). An adjusted total BDI‐II score, one that did not include item 16 (i.e., changes in sleep), served as the dependent variable in this study.

### 2.4 Analyses

IBM SPSS 24 statistical software was used to analyze these data. One‐way analyses of covariance (ANOVA) and chi‐squared tests were used to assess group differences in demographic and health features when variables were normally distributed or categorical, respectively. Sleep variables, MMSE, BDI‐II, average number of medical conditions, and average number of medications were not normally distributed within each group (Kolmogorov‐Smirnov < .05); Mann–Whitney *U* test was used to assess group differences in these variables (Table 1). In the total sample, Spearman's correlation coefficient was used to determine whether different types of sleep disturbances were associated with each other, depression symptom severity, or sex. Spearman's correlation coefficient was also used to determine whether sleeping more or less than usual, compared to no changes, was associated with depression symptoms in each group. Because previous research found an association between long TST (≥ 9 hr) with depression in PD and because long TST was rare in our sample, the four individuals with TST ≥ 9 hr were excluded from analyses used to investigate the association between TST and depression symptoms severity. To test whether the strength of the correlation between sleep disturbances (TST or ISI) and depression severity were different in patients with PD than MC, we conducted Fisher's *Z* transformation for Spearman's correlation coefficient (Snedecor & Cochran, 1980). Sensitivity analyses, with these extreme cases excluded, were conducted to test whether outliers influenced the results. We also conducted sensitivity analyses excluding individuals using antidepressants or sleep aids.

A follow‐up moderation analysis using the PROCESS plugin for SPSS (Model 1) was conducted to test for main effects of ISI and TST and their interactions on depression severity: BDI‐II adjusted total score was the dependent variable; TST and ISI were the independent variables.

## 3 RESULTS

Table 1 shows the results of group comparison analyses for demographic, health, and sleep features. Compared to MC, patients with PD had poorer sleep quality on the PSQI, higher rates of clinically significant sleep disturbance, endorsed about five more symptoms of depression on average, were more likely to report sleeping more than usual on the BDI‐II sleep item, had higher ISI total scores, and took more medications including more antidepressants. The groups did not differ in self‐reported TST.

Shorter TST was associated with greater ISI in both MC and PD, *r*_*s*_(48) = −0.43, *p *=* *.003, *r*_*s*_(50) = −0.67, *p *<* *.001, respectively. Shorter TST was associated with reports of sleeping less than usual in both MC and PD, *r*_*s*_(44)_ _= −0.52, *p *<* *.001, *r*_*s*_(34) = −0.35, *p *=* *.044. Female MC had higher ISI scores than male MC, *r*_*s*_(48) = 0.30, *p *=* *.036. There were no other significant associations between sex and sleep in the total sample or either group. Shorter TST, sleeping more than usual or less than usual, and higher ISI were associated with depression severity in the total sample, *r*_*s*_(94) = −0.35, *p *=* *.001; *r*_*s*_(71) = 0.51, *p *<* *.001; *r*_*s*_(78) = 0.47, *p *<* *.001; *r*_*s*_(98) = 0.46, *p *<* *.001, respectively.

**Figure 1:**
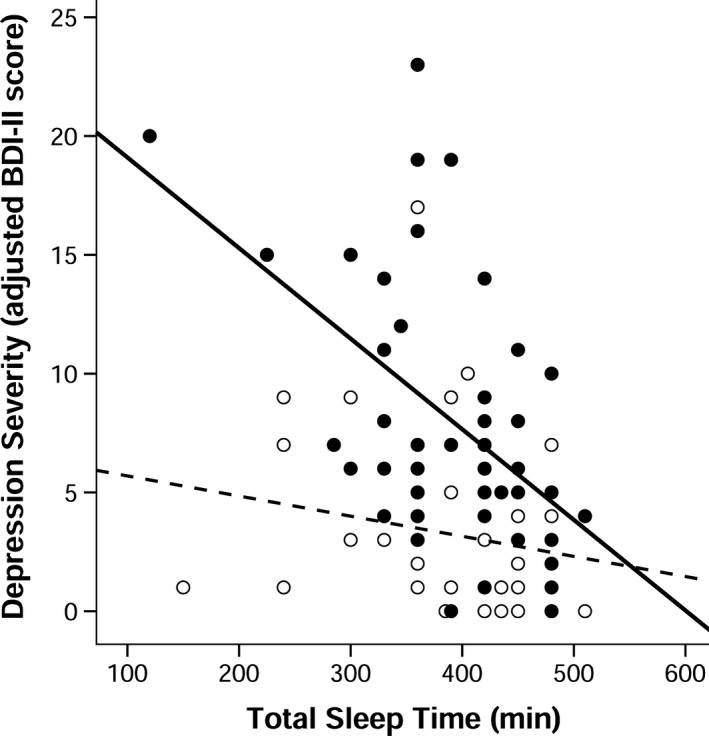
Lower self‐reported total sleep time (TST) on the Pittsburgh Sleep Quality Index was associated with depression severity on the Beck Depression Inventory, 2nd ed. (BDI‐II) among patients with Parkinson's disease (PD) but not among matched controls (MC), *r*_*s*_(47) = −0.47, *p *=* *.001; *r*_*s*_(47) = −0.04, *p *=* *.769, respectively. Fisher's test showed that the correlation between TST and adjusted BDI‐II score was stronger in the PD group than the MC group, *Z* = −2.21, *p *=* *.027. Adjusted BDI‐II total scores did not include item 16 (i.e., sleep changes). Closed circles and black line represent patients with PD; open circles and dotted line represent MC

Figure 1 shows the association between TST and depression severity in PD and MC. There was a significant negative correlation between TST and depression severity in the PD group, but not the MC group, *r*_*s*_(47) = −0.47, *p *=* *.001; *r*_*s*_(47) = −0.04, *p *=* *.769, respectively. Shorter TST was more strongly correlated with depressive symptoms among patients with PD than it was among MC, *Z = *−2.21, *p = *.027. There were more men in our sample, consistent with the prevalence rates of PD. Adding sex to the model did not alter the pattern of significance of the results, nor was it a significant predictor of depression severity. Two TST values (1 PD, 1 MC) and one BDI‐II adjusted total score (1 MC) were statistical outliers (extreme values >/< interquartile range × 2.2 ±  interquartile range). Removing outliers or participants using antidepressants did not alter the pattern of results or level of significance in sensitivity analyses. Removing participants using a sleep aid in sensitivity analyses changed the group difference in correlation strength to a trend only, *p = *.083, but the pattern of results and the correlation between TST and depression severity remained significant in the PD group.

**Figure 2:**
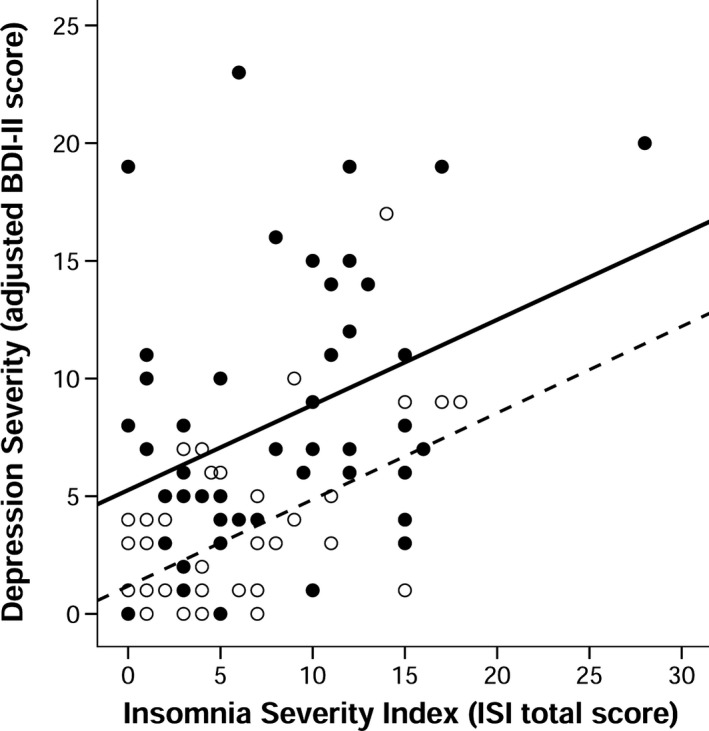
The association between Insomnia Severity Index (ISI) total score and depression severity on the Beck Depression Inventory, 2nd ed. (BDI‐II) in patients with Parkinson's disease (PD) and matched controls (MC)**.** Insomnia severity was significantly associated with depression severity in both groups, *r*_*s*_(50) = 0.33, *p *=* *.020; *r*_*s*_(48) = 0.42, *p *=* *.003, respectively. Adjusted BDI‐II total scores did not include item 16 (i.e., sleep changes). Closed circles and black line represent patients with PD; open circles and dotted line represent MC

Figure 2 shows the association between insomnia severity and depression severity in PD and MC. Insomnia severity was significantly associated with depression severity in both PD and MC, *r*_*s*_(50) = 0.33, *p *=* *.020; *r*_*s*_(48) = 0.42, *p *=* *.003, respectively. The association between ISI and depression severity was not different across groups, *Z *=* *−0.49, *p *=* *.624. Removing outliers or participants using antidepressants did not alter the pattern of results or the significance levels within each group. Removing individuals using a sleep aid reduced the significant association between ISI and depression severity in the PD group to a trend only, *p = *.063, but remained significant in the MC group.

Moderation analysis within the PD group found no significant interaction between ISI and TST in predicting depression severity. With both ISI and TST included in the model, only shorter TST was a significant predictor of depression severity in PD.

## 4 DISCUSSION

In this study, we investigated whether the association between sleep disturbance and depression severity is greater in patients with PD than in MC; we hypothesized that the association would be stronger in patients with PD. We found that shorter TST was associated with greater depression severity in patients with PD than in MC. The rates and severity of sleep disturbance in our PD sample were comparable to previous reports using the PSQI, with over half (58%) reporting sleep disturbance (Borek et al., 2006; Ferreira et al., 2006; Ratti et al., 2015). Consistent with previous studies, patients with PD had more severe insomnia, higher rates of sleeping more than usual, and more symptoms of depression than MC (Ferreira et al., 2006; Tandberg et al., 1998). The absence of a group (PD vs. MC) difference in TST is consistent with previous reports (Happe et al., 2001; Ratti et al., 2015). There is a compelling bivariate association between insomnia and depression severity in PD (Chung et al., 2013; Rutten et al., 2017; Suzuki et al., 2009). Although insomnia and TST were associated with each other and with depression severity, insomnia severity was not more strongly associated with depression in PD than it was in MC in this present study. When TST and ISI were considered together, it was shorter TST that accounted for the majority of the variance in depression severity.

One possible mechanism through which shorter TST may be associated so strongly with depression severity in PD relates to dopamine dysfunction. Interestingly, shorter TST was not associated with sleeping less than usual reported in patients with PD, suggesting the greater association between short TST and depression in PD may be more associated with persistent sleep loss rather than recent changes. In normal individuals, dopamine levels increase in the brain following sleep loss, a putative compensatory mechanism (Drummond et al., 1999; Volkow et al., 2009). Dopaminergic dysfunction associated with PD pathology may reduce these patients’ ability to compensate for the effects of sleep loss, thereby increasing risk for greater depression severity. The cross‐sectional design of this study prevents us from establishing causal relationships or establish why shorter TST is more strongly associated with depression severity in PD than MC. Nevertheless, the results of this study highlight the importance of considering TST as a potential predictor, marker, or consequence of depression severity in these patients.

These findings must be interpreted in the context of several limitations. Assessment of sleep was limited to standardized self‐report measures. Because depression severity was also measured with self‐report, we cannot rule out the possibility that a common factor of self‐report was involved in these findings. However, such an explanation cannot explain the unique association between shorter TST and depression severity in PD compared to MC, an association that was not observed for insomnia severity and depression severity in PD. The absence of a sleep‐related impairment measure specific to PD would have strengthened this study and allowed for comparison with previous reports. Future studies are needed to determine whether PD‐specific sleep measures and objective measures of sleep duration yield similar results. Our relatively small sample size was another limitation of this study; the patients with PD in this study tended to be in the earlier stages of the disease compared to several other studies reported in the literature that did not screen for dementia and sleep disorders other than insomnia (i.e., apnea). In addition, they were relatively young for their disease duration—but within ranges reported previously (Avidan et al., 2013; Naismith et al., 2010)—suggesting that as a group they may have a less severe presentation. Therefore, these results cannot be broadly generalized to patients with PD with greater disease severity. Given the high degree of heterogeneity within patients with PD in terms of etiology, symptom constellations, severity, and so forth, it is possible that sleep vulnerabilities may differ depending on sample characteristics. This line of research could be conducted on selected samples of patients with PD or could be investigated in a larger sample that included more heterogeneity in PD symptoms and stages.

## 5 CONCLUSION

James Parkinson (1817) identified sleep disturbance as an important characteristic of PD. In clinical practice and research, sleep disturbances associated with PD have been overshadowed by the cardinal motor symptoms of the disease. Over the past several decades, studies on sleep in PD have raised awareness that sleep disturbances are strongly associated with depression in this patient sample. This study adds to the growing literature aimed at elucidating the sleep features that may be most strongly associated with depression in PD. The findings from this study suggest that self‐reported short TST may be an important marker, predictor, or consequence of depression severity in patients with PD and may be more strongly associated with depression in PD than among adults without PD. Follow‐up research on the mechanisms of this association is merited. Many PD sleep questionnaires do not assess TST; adding this dimension to sleep assessment may help guide research and clinical practice toward treatments capable of improving depression severity and sleep in these patients.

## CONFLICT OF INTEREST

Dr. Kay was funded by National Institute of Aging (PI: Michael Marsiske, T‐32 AG020499) during the collection, cleaning, and management of these data. He is currently funded by an internal grant from the Office of Research and Creative Activity at Brigham Young University: The Mentoring Environment Grant, titled “Multimodal Neuroimaging of Insomnia during Non‐Rapid Eye Movement Sleep (MNI_NREM).” He receives salary support from Brigham Young University and consulting wages from Draper Psychological Services. The Beck Depression Inventory, 2nd ed. and the Mini‐Mental State Examination were purchased at a reduced rate through dissertation research awards obtained from Pearson Education Inc. (London, UK) and Par Inc. (Lutz, FL, USA), respectively. Dr. Tanner is currently funded by National Institute of Neurological Disorders and Stroke (Co‐I: R01 NS082386), National Institute of Nursing Research (Co‐I: R01 NR014810), and the National Institute of Aging (Co‐I: R01 AG054370). Dr. Bowers receives current funding from State of Florida Education and Ethel Moore Alzheimer's Program, the National Institutes of Health (PI/MPI: T32 NS082168), National Institute of Aging (PI/MPI: R21 AG057200), National Institute of Mental Health (Co‐I: R03 MH109336), National Institute of Neurological Disorders and Stroke (Co‐I: R01 NS096008; Co‐I: R01 NS082386; Co‐I: UH3 NS095553), and National Institute of Child Health Development (Co‐I: R01 HD091658). She also receives additional salary support from UF. The authors received no other support for this study and have no conflicts of interest.
